# Elucidation of ligninolysis mechanism of a newly isolated white-rot basidiomycete *Trametes hirsuta* X-13

**DOI:** 10.1186/s13068-021-02040-7

**Published:** 2021-09-25

**Authors:** Jiangshan Ma, Qiang Li, Yujie Wu, Huimin Yue, Yanghong Zhang, Jiashun Zhang, Muling Shi, Sixian Wang, Gao-Qiang Liu

**Affiliations:** 1 Hunan Provincial Key Laboratory of Forestry Biotechnology, Central South University of Forestry and Technology, Changsha, 410004 Hunan People’s Republic of China; 2 International Cooperation Base of Science and Technology Innovation On Forest Resource Biotechnology, Central South University of Forestry and Technology, Changsha, 410004 Hunan People’s Republic of China

**Keywords:** Lignin degradation, White-rot basidiomycete, *Trametes hirsuta* X-13, 2D-HSQC NMR, Cleavage, Interunit linkages

## Abstract

**Background:**

Lignin is a complex aromatic heteropolymer comprising 15–30% dry weight of the lignocellulose. The complex structural characteristic of lignin renders it difficult for value-added utilization. Exploring efficient lignin-degrading microorganisms and investigating their lignin-degradation mechanisms would be beneficial for promoting lignin valorization. In this study, a newly isolated white-rot basidiomycete, *Trametes hirsuta* X-13, with capacity to utilize alkaline lignin as the sole substrate was investigated.

**Results:**

The analysis of the fermentation properties of *T*. *hirsuta* X-13 using alkaline lignin as the sole substrate, including the mycelial growth, activities of ligninolytic enzymes and the rates of lignin degradation and decolorization confirmed its great ligninolysis capacity. The maximum lignin degradation rate reached 39.8% after 11 days of *T*. *hirsuta* X-13 treatment, which was higher than that of reported fungi under the same condition. Fourier transform infrared spectrometry (FTIR), gas chromatography–mass spectrometry (GC–MS) scanning electron micrographs (SEM), two-dimensional heteronuclear single quantum coherence NMR analysis (2D-HSQC NMR) collaborated with pyrolysis gas chromatography–mass spectrometry (py-GC/MS) analyses proved that lignin structure was severely deconstructed along with amounts of monomer aromatics generated. Furthermore, according to those chemical analysis, in addition to canonical C_α_–C_β_ breakage, the cleavage of lignin interunit linkages of β–β might also occur by *T*. *hirsuta* X-13.

**Conclusions:**

This study characterized a newly isolated white-rot basidiomycete *T*. *hirsuta* X-13 with impressive alkaline lignin degradation ability and provided mechanistic insight into its ligninolysis mechanism, which will be valuable for the development of lignin valorization strategies.

**Supplementary Information:**

The online version contains supplementary material available at 10.1186/s13068-021-02040-7.

## Background

Lignin is a complex aromatic and optically inactive amorphous heteropolymer accounting for 15–30% dry weight of the lignocellulosic biomass, which is the most abundant source of renewable aromatic carbon on earth. It contains three different phenyl propane units (*p*-hydroxyphenyl, guaiacyl and syringyl units) connected by a multiplicity of C–O and C–C bonds, such as β-O-4, β–β, 4-O-5, β-5 [1]. The heterogeneous and complex structural characteristics of lignin render it difficult for degradation. In lignocellulosic biomass structure, lignin is embedded between cellulose and hemicellulose structures, forming a complex heterogeneous network that limits the accessibility of enzymes or chemicals [2]. Hence, depolymerization of lignin structure of lignocellulosic biomass is a crucial step in the biorefinery industry aiming at increasing the accessibility of relevant enzymes to polysaccharides [3]. In addition, lignin consist of phenyl propane units also representing a potentially intriguing valuable source of renewable aromatic chemicals [4, 5]. The valorization of lignin by microorganisms which converted it into valuable chemicals such as vanillin, eugenol and other phenolics has attracted much attention [6–8].

To date, a number of microorganisms including fungi and bacteria were found with capacity to depolymerize lignin [1, 9]. In fungi, as the extensively studied lignin degraders, white-rot basidiomycetes were demonstrated as the exclusive species that can degrade lignin completely [10, 11]. White-rot basidiomycete fungi produce several types of highly efficient and unique extracellular oxidative enzymes including laccase, and lignin-degrading peroxidases that are involved in lignin degradation. Instead of degrading lignin, brown-rot fungi were found to modify the lignin structure through hydroxyl radical oxidants produced via Fenton chemistry pathway [12, 13]. Although some bacteria, such as *Pseudomonas putida*, *Cupriavidus basilensis*, and *Rhodococcus jostii* have been reported with lignin degradation ability, their lignin depolymerization activities were significantly weaker than those of fungi [14–16]. Hence, ligninolytic fungi especially white-rot basidiomycete fungi represent promising microorganisms for lignin biological treatment.

Although ligninolytic enzymes have been extensively identified and characterized in white-rot fungi, the degradation pathways of lignin largely remain unknown. The only demonstrated ligninolysis routes in white-rot fungi were C_α_–C_β_ and β-O-4 cleavages based on the detection of the corresponding benzoic acid derivatives during the lignocellulosic biomass degradation [17–20]. Furthermore, knowledge of the degradation routes of lignin by white-rot basidiomycete fungi when used as the sole carbon source were less studied. With the global availability and chemical versatility, lignin is regarded as an intriguing renewable aromatic complex and has a promising potential in the production of commercially valuable chemicals [21]. Yet so far the use of enzymatic technology for commercial lignin conversion remains challenging. Exploring efficient lignin-degrading fungi and investigating their degradation mechanisms for lignin substrate would be available for promoting lignin valorization.

In this study, we characterized a newly isolated ligninolytic white-rot basidiomycete *Trametes hirsuta* X-13. To assess the lignin degradation ability, the fermentation properties of *T*. *hirsuta* X-13 using alkaline lignin as the sole carbon source including the mycelial growth, activities of lignin-degrading enzymes and rates of lignin degradation and decolorization were investigated. Furthermore, Fourier transform infrared spectrometry (FTIR), gas chromatography–mass spectrometry (GC–MS), scanning electron micrographs (SEM), two-dimensional heteronuclear single quantum coherence NMR analysis (2D-HSQC NMR) and pyrolysis gas chromatography–mass spectrometry (py-GC/MS) were employed for exploration of the structure variations of lignin residues, the corresponding metabolic compounds and the ligninolysis route by the strain.

## Results and discussion

### Isolation, screening and identification of ligninolytic fungus strain

**Fig. 1:**
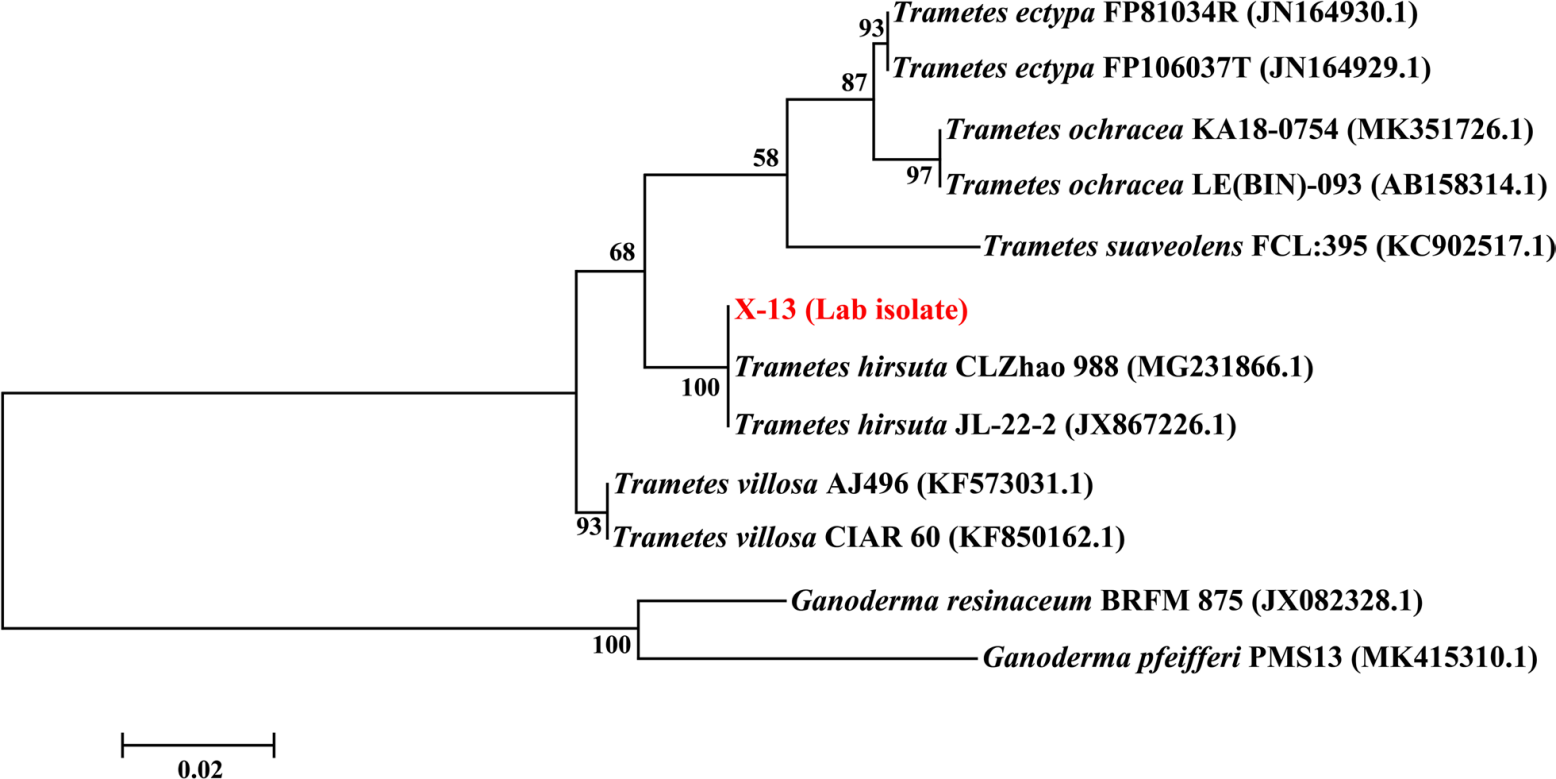
Phylogenetic tree presents the relationship of strain X-13 with other related fungal strains based on ITS region sequences. The phylogenetic tree was constructed by the maximum likelihood algorithm (1000 bootstrap trials)

During the isolation process, 13 strains in total were isolated from collected rotten wood samples. Of these isolates, the strain X-13 produced the largest colorization zone and decolorization zone on guaiacol-containing and Azure B-containing PDA medium, respectively, in the screening procedure (Additional file 1: Figure S1). Guaiacol and Azure B are commonly used as indicators for determining the lignolytic potential [22]. This indicated the potentially strong ligninolytic activity of strain X-13. The morphological analysis of strain showed that the colony of strain X-13 was milky white with the dense hyphae, and it revealed a thread-like septate mycelium without any reproductive structures (Additional file 2: Figure S2), which were similar to those of reported *Trametes hirsuta* genus strains [23, 24]. Moreover, the ITS region sequence of strain X-13 (GenBank: MT995079) showed 99% similarity with that of white-rot basidiomycete *Trametes hirsuta* JL-22-2 according to NCBI BLAST algorithm analysis. As shown in Fig. 1, phylogenetic tree analysis revealed that strain X-13 belongs to species of *Trametes hirsuta*. Therefore, the strain X-13 was identified as *Trametes hirsuta* strain, which has since been deposited in the China General Microbiological Culture Collection (CGMCC No. 18567).

### Characterization of lignin degradation by *T*. *hirsuta* X-13

**Fig. 2:**
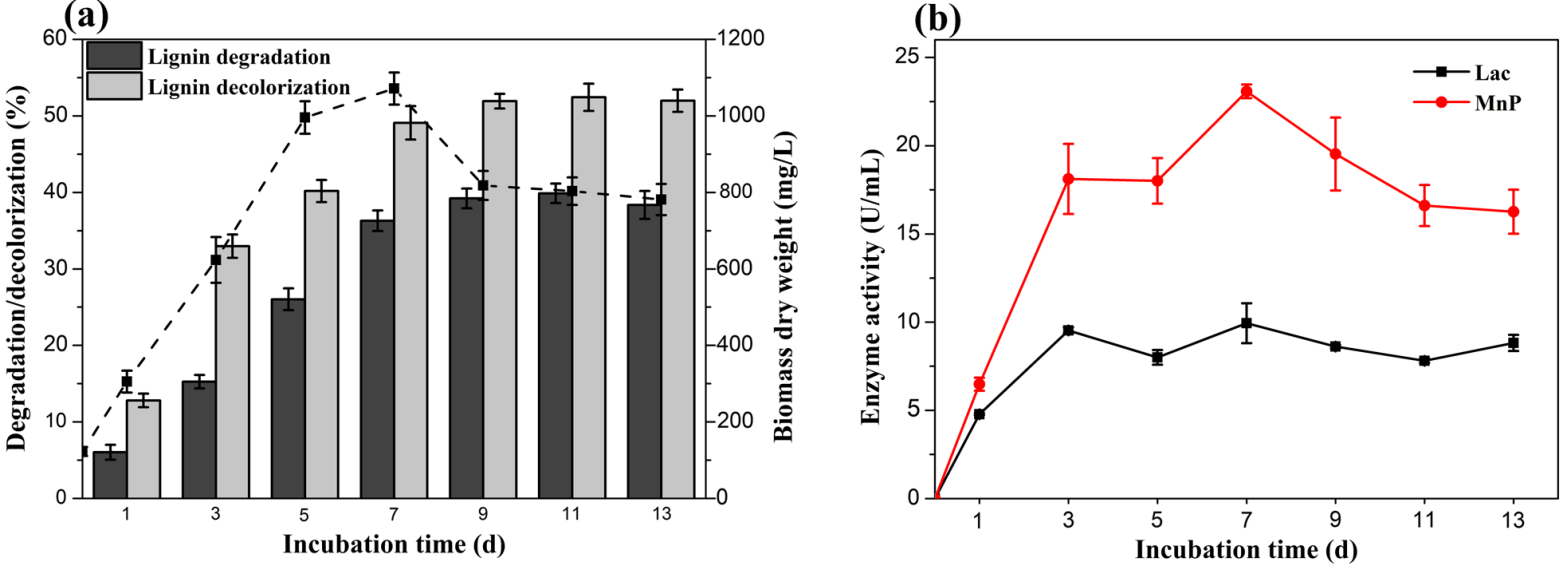
The variations of lignin degradation and decolorization, mycelial biomass growth (**a**) and ligninolytic enzymes activities (**b**) during the *T*. *hirsuta* X-13 incubation with lignin as the sole carbon source

To evaluate the lignin degradation property of *T*. *hirsuta* X-13, the growth of mycelial biomass, the rates of lignin degradation and decolorization, and the ligninolytic enzymes activity were investigated during the incubation with alkaline lignin as sole carbon source. As shown in Fig. 2a, the dry weight of mycelial biomass increased with the time and achieved a maximum on day 7 and then started to decline gradually. This suggested that *T*. *hirsuta* X-13 could utilize alkaline lignin as the sole carbon and energy source for growth. Although numerous microorganisms including fungi and bacteria have been found with lignin degradation ability, less was reported with sole lignin substrate for growth [10, 11]. As the complete oxidation of lignin is highly exothermic, lignin degradation is too slow to function as a source of metabolic energy [25]. Hence, during the lignin degradation process of the most ligninolytic microorganisms, additional carbon or nitrogen source was needed to be supplemented to initiate lignin depolymerization [25–27]. Despite that the presence of polysaccharides impurities in the alkaline lignin might also contribute to the growth of *T*. *hirsuta* X-13, the small amount of these (1.52%) (Additional file 3: Table S1) limited their effects on the strain growth. The rapid growth of *T*. *hirsuta* X-13 in the medium containing alkaline lignin as the sole carbon source suggested the dramatic capacity of lignin depolymerization and metabolism for this fungus strain. The decline of fungal growth after 7 days of incubation in lignin-containing medium might be due to the accumulation of toxic metabolic compounds including lignin-derived aromatic compounds and the reduction of nutrient essential for strain growth.

The rates of lignin degradation and decolorization showed the same trends during whole incubation time (Fig. 2a). The rates of both lignin degradation and decolorization increased rapidly in the initial 7 days, which then almost remained unchanged. This was directly correlated with the mycelial biomass growth of the strain. The maximum rates of both lignin degradation and decolorization were obtained at 11th day, with the values of 39.8% and 52.4%, respectively. The maximum lignin degradation rate by *T*. *hirsuta* X-13 was higher than that of reported fungi under the same condition [11]. These results further indicated the strong ability of lignin degradation by *T*. *hirsuta* X-13.

The degradation of lignin by white-rot fungi mainly depends on the action of a series of ligninolytic enzymes including laccase, lignin peroxidase and manganese peroxidase [28]. As shown in Fig. 2b, the activities of laccase (Lac) and manganese peroxidase (MnP) showed approximate increase trend during the initial 7 days. After that, they started to decline. The lignin peroxidase (LiP) activity was undetected across the whole incubation time. Furthermore, secretomic analysis was carried out to provide insight into the ligninolytic system of *T*. *hirsuta* X-13. There were in total 792 proteins identified in lignin cultures of *T*. *hirsuta* X-13 (Additional file 4), which were divided into six categories according to their biological function (Additional file 5 and Additional file 6: Figure S3). Among these, 42 proteins that functioned as ligninolytic enzymes are listed in Additional file 7: Table S2, which include six laccases, eight manganese peroxidases, four lignin peroxidases, two dye-decolorizing peroxidases and one GMC oxidoreductase. Most of those identified ligninolytic enzymes were predicted as the extracellular proteins. The existence of the abundance of those ligninolytic enzymes cocktail in the secretory proteins of *T*. *hirsuta* X-13 indicated its strong ability towards lignin degradation. Although four lignin peroxidases were found, the corresponding activity was undetectable during the lignin degradation by *T*. *hirsuta* X-13, which might be due to that the protein level of lignin peroxidase was too low to be detected for enzyme activity. It should be noted that considerable amount of proteins (71) belonging to CAZy (glycoside hydrolase, carbohydrate esterase and carbohydrate-binding module) were found in the secretory proteins. As lignin was used as the sole carbon with less impurity, those identified CAZy enzymes might be derived from the constitutive expression by the strain with low level [29, 30]. Quantitative secretomic analysis is needed in further study to clarify this speculation. In addition, the variation of detected ligninolytic enzymes activity was in line with that of strain growth and lignin degradation. These results suggested that laccase and manganese peroxidase were the main enzymes responsible for lignin degradation by *T*. *hirsuta* X-13. A number of *Trametes* genus strains have been found with lignin-degrading activity, and they could secrete abundance of lignin-degrading enzymes including laccase, lignin peroxidase and manganese peroxidase [31, 32]. However, the lignin degradation studies with these strains were conducted under the co-existence of a large amount of glucose substrate [33, 34]. The utilization of alkaline lignin as the sole carbon resource by *Trametes hirsuta* strain was rarely reported.

### FTIR analysis

**Fig. 3:**
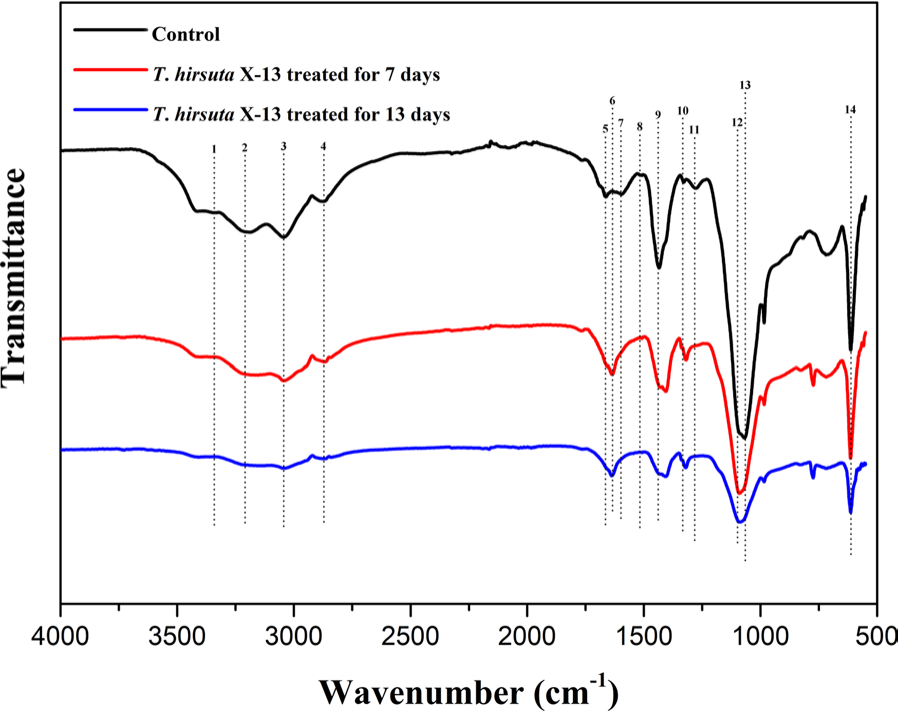
FTIR spectra of the control and treated lignin samples with *T*. *hirsuta* X-13 for 7 and 13 days. The labeled peaks with number assigned to the corresponding functional groups and structures are listed in Table 1

To investigate the changes of functional groups and chemical structures of alkaline lignin after treatment with *T*. *hirsuta* X-13, FTIR analysis was performed. As shown in Fig. 3, the FTIR spectra of raw and treated lignin by *T*. *hirsuta* X-13 displayed distinct changes, especially on the lignin fingerprint region (1700–1000 cm^−1^). The major peaks of samples were assigned as listed in Table 1 based on the previous literature reports [35–37].

**Table 1:** Functional groups and structures assignment of the FTIR spectra peaks

Number	Wavenumber (cm^−1^)	Functional groups and structures assignment
1	3420	OH stretching vibration
2	3210	OH stretching vibration
3	3050	OH stretching vibration
4	2860	C–H stretching in aromatic methoxyl groups
5	1650	Absorbed O–H and conjugated C–O
6	1635	C=C stretching vibration peak in benzene ring
7	1600	Aromatic skeletal vibrations and C=O stretch; *S* > *G*
8	1511	Aromatic skeletal vibrations; *G* > *S*
9	1420	Aromatic skeletal vibrations combined with O–CH_3_ in plane deform
10	1317	C–O in syringyl group
11	1275	C–O in guaiacyl group
12	1090	C–O vibrations in aliphatic ethers and/or in secondary alcohols
13	1041	C–O in guaiacyl group
14	618	Stretching vibrations of the C–S bond linked to the aromatic ring

The intensities of bands at 2860 cm^−1^ and 1420 cm^−1^ which were assigned to C–H stretching in aromatic methoxyl groups and aromatic skeletal vibrations combined with O–CH_3_ in plane deform, respectively, were reduced with the increase in the treatment time of *T*. *hirsuta* X-13, suggesting the demethoxylation reaction of lignin by the strain. The decline of the intensities of the bands at 1275 cm^−1^ and 1041 cm^−1^ ascribed to C–O in guaiacyl group, and 1511 cm^−1^ belongs to aromatic skeletal vibrations (G > S) in *T*. *hirsuta* X-13-treated samples implied the removal of guaiacyl unit in lignin. Meanwhile, the weakened intensities of bands at 1600 cm^−1^ and 1317 cm^−1^, relating to aromatic skeletal vibrations and C=O stretch (S > G) and C–O in syringyl group, respectively, suggested the decline of syringyl unit content in lignin after *T*. *hirsuta* X-13 treatment. The decreased intensities of the bands at 1090 cm^−1^ corresponding to C–O vibrations in aliphatic ethers and/or in secondary alcohols in *T*. *hirsuta* X-13-treated samples illustrated that more easily degradable materials were produced after the demethylation and side chain oxidation of lignin. In addition, the reduction of intensity of the bands at 618 cm^−1^ ascribed to stretching vibrations of the C–S bond linked to the aromatic ring indicated a side chain oxidation of lignin. These significant variations of FTIR spectra on *T*. *hirsuta* X-13-treated lignin relative to control indicated that lignin structure was largely destructed by this fungus strain, which further demonstrated its impressive ligninolytic capacity.

### GC–MS analysis

To investigate the degradation products from alkaline lignin by *T*. *hirsuta* X-13, GC–MS analysis was carried out. The low molecular weight metabolic compounds identified from the peaks of the total ion chromatograms (Additional file 8: Figure S4) are listed in Table 2. A total of 12 aromatics and 3 fatty acids were identified from the lignin samples after 13 days of *T*. *hirsuta* X-13 treatment, and only 4 aromatics were obtained from the control group. Among these aromatic compounds, 2,4-dihydroxybenzaldehyde, gentisic acid, 2,6-dihydroxybenzoic acid, 3,4,5-trihydroxybenzoic acid, 3,5-ditert-butylphenol, 4,5-dihydroxyphenylacetic acid, 2-hydroxybenzoic acid, 3,5-dimethoxy-4-hydroxyacetophenone, 4‑hydroxyphenylacetic acid, 3,4-dihydroxyphenylacetic acid were only observed in *T*. *hirsuta* X-13-treated sample, suggesting the occurrence of lignin depolymerization by the strain treatment. Meanwhile, the presence of three long-chain fatty acid compounds, i.e., dodecanedioic acid, 9,12,15-octadecatrienoic acid and hexadecanoic acid in *T*. *hirsuta* X-13-treated sample might indicate that the generated aromatic compounds derived from lignin structure were further converted to fatty acid compounds via aromatic ring cleavage by the strain [38, 39]. However, further studies are needed to elucidate the metabolic pathway of fatty acids during the lignin degradation by *T*. *hirsuta* X-13. In addition, the predomination of aromatic acid compounds in the identified products implied the splitting of the aliphatic side chains of lignin units by *T*. *hirsuta* X-13. These suggested that the lignin structure was deeply destructed by the strain, which was consistent with FTIR analysis results.

**Table 2:** Identification of degradation products from the control and *T*. *hirsuta* X-13-treated lignin cultures by GC–MS method

RT (min)	Compound	Control	Treated
8.10	2,4-Dihydroxybenzaldehyde	−	+
8.78	Gentisic acid	−	+
11.06	Phenylacetic acid	+	+
12.40	2,6-Dihydroxybenzoic acid	−	+
12.66	Dodecanedioic acid	−	+
13.99	3,4,5-Trihydroxybenzoic acid	−	+
14.20	9,12,15-Octadecatrienoic acid	−	+
14.83	3,5-Ditert-butylphenol	−	+
14.95	Acetovanillone	+	−
16.00	4,5-Dihydroxyphenylacetic acid	−	+
16.21	4-Hydroxybenzaldehyde	+	−
17.39	2-Hydroxybenzoic acid	−	+
17.76	Vanillin	+	−
17.84	3,5-Dimethoxy-4-hydroxyacetophenone	−	+
19.47	Hexadecanoic acid	−	+
19.92	4‑Hydroxyphenylacetic acid	−	+
20.60	4,6-Dihydroxybenzoic acid	+	+
24.07	3,4-Dihydroxyphenylacetic acid	−	+

It is noticeable that there were three unique aromatic compounds, i.e., acetovanillone, 4-hydroxybenzaldehyde and vanillin identified in the control group. These three aromatic compounds were the canonical aromatic metabolites presented in bacterial lignin degradation process [15, 25, 40, 41]. Moreover, vanillin generated from C_α_–C_β_ oxidative cleavage of β-aryl ether components of lignin was the major metabolite in lignin degradation process of many lignin-degrading microorganisms [1]. The presence of these three aromatic compounds in the control group suggested that the partial oxidation and degradation of lignin occurred during the production process [15, 37]. While the absence of these three aromatic compounds in lignin-derived metabolites of *T*. *hirsuta* X-13-treated group indicated the catabolism of these compounds by the strain for growth.

Acetovanillone could be converted to ferulic acid or vanillin by C–C coupling reaction, while this compound was found to be resistant for some bacteria [42]. That acetovanillone and its intermediate metabolites (ferulic acid and vanillin) were not detected suggested the strong degradation and metabolism capacity of *T*. *hirsuta* X-13 for lignin and its aromatic units. The appearance of 4-hydroxyphenylacetic acid corresponding with the absence of 4-hydroxybenzoic acid implied that C_α_–C_β_ cleavage might exist during the 4-hydroxybenzoic acid degradation by *T*. *hirsuta* X-13 [43]. It has been reported that the C_α_–C_β_ cleavage for lignin or aromatic compounds could be catalyzed by laccase [37, 44]. This was in line with the above results of enzymatic analysis that laccase activities were detected across the whole process of lignin degradation by *T*. *hirsuta* X-13.

### SEM analysis

**Fig. 4:**
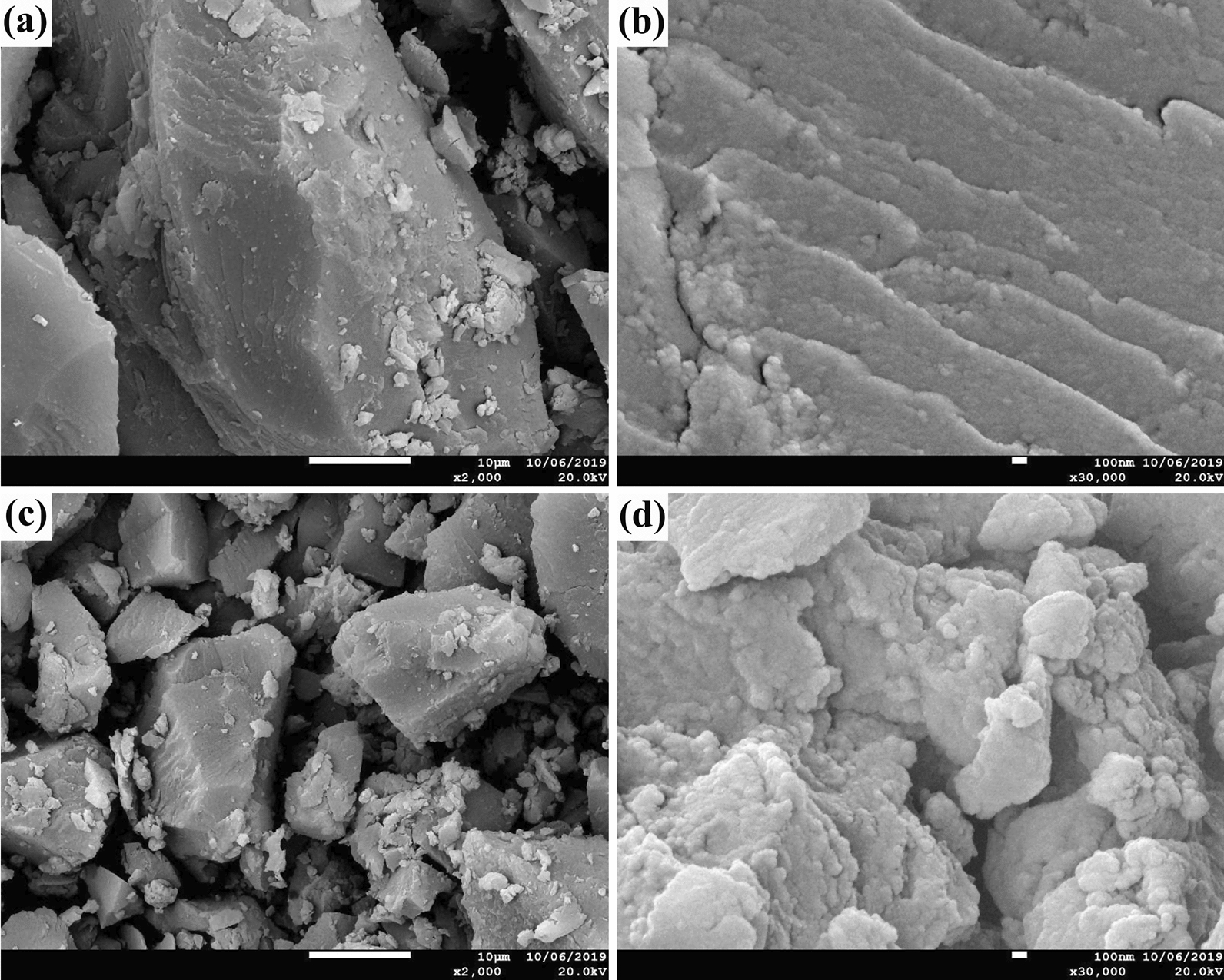
Scanning electron micrographs of the control (**a**, 2000 × and **b**, 30,000×) and treated lignin samples by *T*. *hirsuta* X-13 for 13 days (**c**, 2000× and **d**, 30,000×)

To investigate the morphological changes of alkaline lignin after degraded by *T*. *hirsuta* X-13, SEM analysis was performed to observe the surface microstructure of lignin samples. As shown in Fig. 4, the surface structure of control lignin samples and after *T*. *hirsuta* X-13 treatment revealed a dramatic distinction. The control lignin samples consist of large fragments with smooth flat surface structure (Fig. 4a, b). However, the particle size of lignin treated by *T*. *hirsuta* X-13 was reduced with smaller fragments generated (Fig. 4c). Moreover, the surface of treated lignin turned rugged with more cracks (Fig. 4d). The remarkable changes of treated lignin particle size and surface also confirmed that the lignin was severely degraded by *T*. *hirsuta* X-13.

### 2D-HSQC NMR characterization

**Fig. 5:**
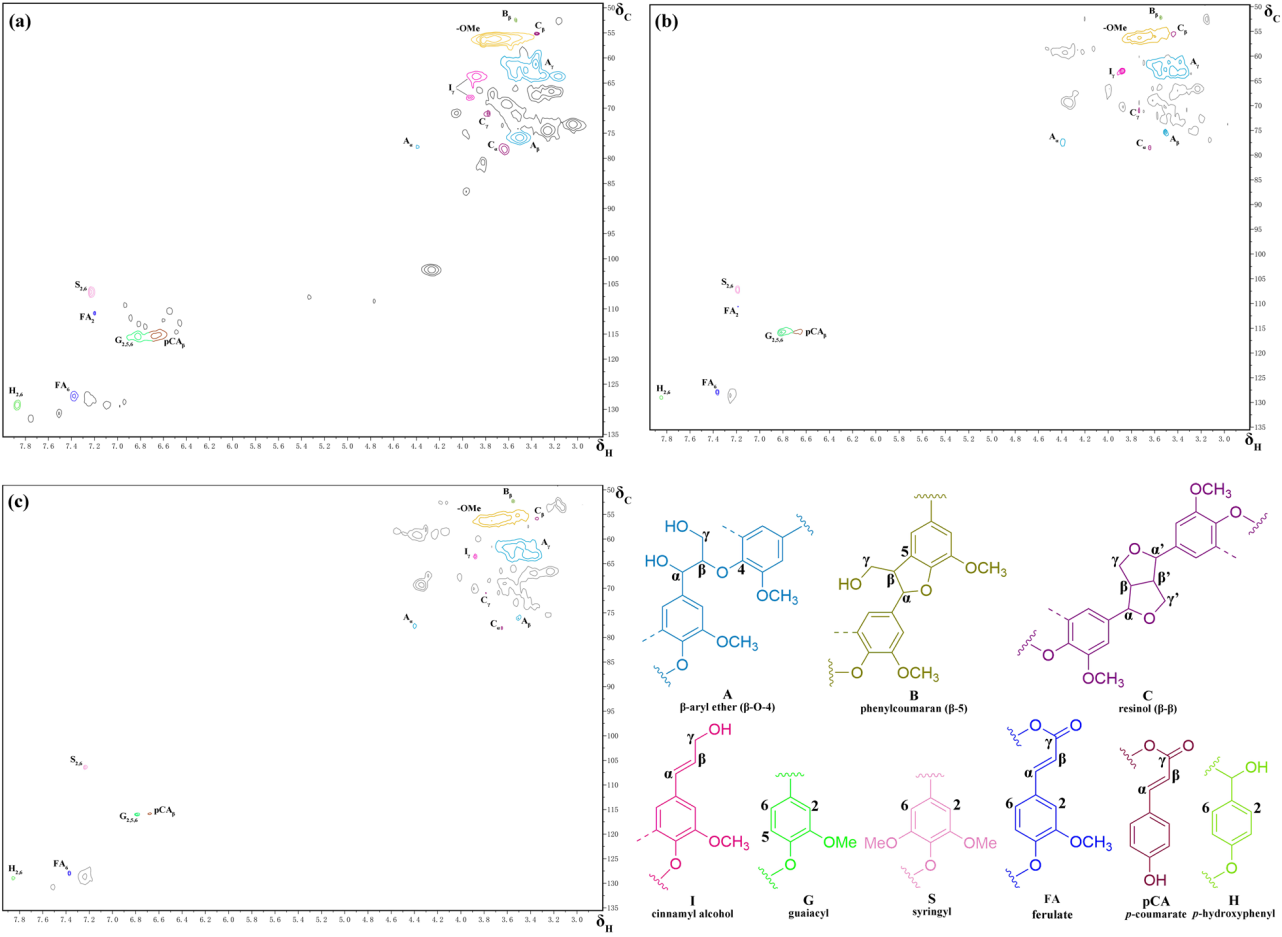
2D-HSQC NMR spectra of the control (**a**) and treated lignin samples after 7 days (**b**) and 13 days (**c**) by *T*. *hirsuta* X-13. Chemical shift assignments are listed in Table 3. The main structures present in lignin: (A) β-aryl ether (β-O-4); (B) phenylcoumaran (β-5); (C) resinol (β–β); (I) cinnamyl alcohol; (G) guaiacyl; (S) syringyl; (FA) ferulate; (pCA) *p*-coumarate; (H) *p*-hydroxyphenyl

To explore the changes in alkaline lignin structure during fungal treatment, 2D-HSQC NMR spectra analysis was employed. The 2D-HSQC NMR spectra of control lignin samples and after 7 and 13 days treatment by *T*. *hirsuta* X-13 are presented in Fig. 5. The correlation peaks of lignin from the spectra are assigned and listed in Table 3. As shown in Fig. 5a, signals from the aliphatic (δ_C_/δ_H_ 80–140/6.0–8.0) and aromatic (δ_C_/δ_H_ 50–80/2.5–6.0) regions of the spectra including methoxyls, β-O-4 (aryl ethers), β–β (resinols) and β-5 (phenylcoumarans) structures, pCA (*p*-coumarates), FA (ferulate), S-, G- and H-type units, were clearly detected in NMR spectra of control samples. The relative abundances of lignin interunit linkages and dominant aromatic units are listed in Table 4. It should be noted that compared to control, the intensities of signals of most of those structures were significantly decreased in the lignin residual after treated by *T*. *hirsuta* X-13 (Fig. 5). This might be due to that the lignin structure was greatly modified after the fungus strain treatment, which might severely impede the solubility of lignin residual in dimethyl sulfoxide.

**Table 3:** Assignments of ^13^C–^1^H correlation signals in the 2D-HSQC NMR spectra from lignin

Signals	δ_C_/δ_H_	Assignments
*B*_β_	52.19/3.54	Phenylcoumaran (B)
*C*_β_	55.98/3.37	C_β_–H_β_ in resinol (C)
MeO	56.23/3.72	C–H in methoxyls
*I*_γ_	63.65–65.41/3.88–3.97	C_γ_–H_γ_ in cinnamyl alcohol end-groups (I)
*A*_γ_	62.48/3.45	C_γ_–H_γ_ in phenylglycerol (A)
*A*_β_	75.89/3.50	C_β_–H_β_ in phenylglycerol (A)
*A*_α_	77.70/4.39	C_α_–H_α_ in phenylglycerol (A)
*C*_γ_	71.34/3.79	C_γ_–H_γ_ in resinol (C)
*S*_2,6_	106.64/7.23	C_2,6_–H_2,6_ in syringyl units (S)
*G*_2_	115.58/6.82	C_2_–H_2_ in guaiacyl units (G)
*G*_5_	115.48/6.81	C_5_–H_5_ in guaiacyl units (G)
*G*_6_	115.49/6.80	C_6_–H_6_ in guaiacyl units (G)
FA_2_	111.14/7.19	C_2_–H_2_ in ferulate (FA)
pCA	115.39/6.67	C–H in *p*-coumarates (pCA)
FA_6_	127.36/7.38	C_6_–H_6_ in ferulate (FA)
H_2,6_	129.17/7.87	C_2,6_–H_2,6_ in *p*-hydroxyphenyl units (H)

**Table 4:** Semiquantitative 2D-HSQC NMR spectra analysis of structural characteristics of lignin

	Control	7 days	13 days
Lignin interunit linkages^a^ (%)			
β-Aryl ether	80.2	87.2	96.0
Phenylcoumaran	1.2	1.3	1.7
Resinol	7.3	3.5	0.7
Cinnamyl alcohol	11.3	8.0	1.6
Total	100	100	100
Condensation degree (β–β/β-O-4)	0.091	0.040	0.007
Lignin aromatic units^b^			
*H* (%)	20.1 (124.6)	11.8 (10.1)	11.9 (6.2)
*G* (%)	55.1 (340.7)	65.4 (56.2)	53.2 (27.8)
*S* (%)	24.8 (153.3)	22.7 (19.5)	35.0 (18.3)
Total	100 (618.6)	100 (85.8)	100 (52.3)
*S*/*G* ratio	0.45	0.35	0.66
*p*-Hydroxycinnamates^c^			
* p*-Coumarate (%)	31.1	23.4	18.6
Ferulates (%)	24.8	15.2	12.4

^a^Relative distribution of lignin interunit linkages

^b^Relative distribution of lignin aromatic units (*H* + *G* + *S* = 100)

^c^*p*-Coumarate and ferulate molar content as percentages of lignin content (*H* + *G* + *S*)

In aliphatic region, as listed in Table 4, the percentage of lignin interunit linkages greatly changed in *T*. *hirsuta* X-13-treated sample relative to control, suggesting that the interunit linkages in lignin might be severely deconstructed during *T*. *hirsuta* X-13 degradation. The condensation degree (β–β/β-O-4) of the lignin were largely decreased with the increment of incubation time of *T*. *hirsuta* X-13, which further confirmed the depolymerization of lignin by *T*. *hirsuta* X-13. In addition, the percentages of resinol (β–β) and cinnamyl alcohol were reduced with the increment of incubation time of *T*. *hirsuta* X-13, indicating that β–β and lignin end-groups were more susceptible to degrade by *T*. *hirsuta* X-13. C–C bonds including β–β were found to be more resistant to microorganism cleavage than ether bonds [45–47]. Though β–β cleavages pathway have been reported in the lignin degradation by *Ceriporiopsis subvermispora*, the used substrate was lignocellulosic biomass wheat straw instead of pure lignin in these study [19, 20]. The complicated composition of biomass might disturb the lignin utilization by the fungi and the polysaccharides components residues might affect the signals of lignin detection in NMR spectra [48, 49]. The employment of lignin as the sole carbon source for exploring the fungus lignin degradation pathway provided a controlled substrate for research. By using alkaline lignin as the sole carbon source, our results showed that β–β cleavage might occur during the lignin degradation process of *T*. *hirsuta* X-13. These results implied that the strain could substantially cleave the highly stable interunit linkage of lignin. Laccase has been found with capacity to break the lignin interunit linkages including β–β [37, 50, 51]. The secretion of laccase by *T*. *hirsuta* X-13 might be responsible for cleavage of this interunit linkage of lignin.

In the aromatic region, the percentage of G-type was more than twice those of S- and H-type units, suggesting that this alkaline lignin belongs to G-rich lignin (Table 4). Compared to control, the percentage of H-type unite in the lignin residual treated by *T*. *hirsuta* X-13 was decreased. While the percentages of G- and S-type units changed with opposite trends during the treatment of *T*. *hirsuta* X-13. The ratio of S/G declined in the lignin residual after degraded by *T*. *hirsuta* X-13 for 7 days relative to control, and the value of which increased from 0.35 up to 0.66 after 13 days treatment. A similar variation trend of S/G ratio was also obtained from the py-GC–MS analysis (Additional file 9: Table S3). The detected S/G ratio in py-GC–MS experiment was lower than that of 2D-HSQC NMR spectra analysis, which might be caused by that the demethylation reaction rate of S-type unit was faster than that of G-type unit in pyrolysis process [52]. This indicated that S-type unit was more susceptible to utilized by *T*. *hirsuta* X-13 in the first 7 days incubation, while it preferentially degraded G-type unit in the last 6 days of incubation. S-type unit was regarded as the most recalcitrant structure toward biodegradation than other two units, as it has two methoxyl groups with lower redox potential [53]. The preferential degradation of recalcitrant structure of S-type unit in the early stage might be attributed to the fast growth of fungus in the first 7 days incubation, which might secrete more relevant enzymes to attack the structure. The reduction of these basic lignin-derived aromatic units might be attributed to the further conversion into other aromatics or fatty acids via demethylation and cleavage of aromatic ring, which then consumed by the strain for growth [54]. The accumulation of certain toxic metabolic compounds derived from these lignin-derived aromatic units might contribute to the decline of fungal growth after 7 days incubation. Further studies focusing on quantitation and tracking of these aromatics are needed to elucidate the metabolism of lignin-derived aromatics by the fungus strain.

## Conclusions

In summary, this study characterized the strong capacity of a newly isolated white-rot basidiomycete *T*. *hirsuta* X-13 for lignin degradation and provided a lignin degradation route insight into its ligninolysis. The maximum lignin degradation rate reached 39.8% after 11 days of treatment with this strain using alkaline lignin as the sole carbon source. Both Lac and MnP activities were observed across the whole incubation time. The employment of chemical analysis elucidated that the structure of lignin residual was severely destructed, and the cleavage of the interunit linkages of lignin including C_α_–C_β_ and β–β occurred in the ligninolysis routes of *T*. *hirsuta* X-13. These results suggested that *T*. *hirsuta* X-13 might be a promising candidate for application on lignin valorization, and the insight of its ligninolysis pathway could broaden the knowledge of lignin degradation mechanisms of fungi.

## Methods

### Ligninolytic fungus strain isolation

Rotten wood samples were collected from the forests of Yuelu Mountain in Changsha city, China. The samples were cut to 0.5 × 0.5 cm and was washed three times using sterile water. Then the samples were placed in the center of Petri dish containing enrichment medium. Potato dextrose broth (PDA) medium contained (g/L) potato extract (200), glucose (10), MgSO_4_ (1.5), KH_2_PO_4_ (3.0), vitamin B (0.05), agar (15) were used as enrichment medium. After cultivation of isolated fungus strains for 2 to 3 generations, the pure isolates obtained were further inoculated in the screen media for ligninolytic strain screening. The screen media included Azure B-containing and guaiacol-containing PDA medium which containing potato dextrose agar (PDA) supplemented with Azure B (0.1 g/L) or guaiacol (0.1 g/L), respectively. The strains produced the largest diameter of the decolorization zone in Azure B-containing PDA medium or colorization zone in guaiacol-containing PDA medium were selected for further study.

### Microorganism identification

The isolated fungus was identified by morphological analysis and internal transcribed region sequence (ITS) fingerprinting. Total DNA of fungus strain was extracted using a Fungi Genomic DNA Extraction Kit (Solarbio, Beijing, China). Amplification of ITS region sequence was performed using ITS 1 forward (TCCGTAGGTGAACCTGCGG) and ITS 4 reverse (TCCTCCGCTTATTGATATGC) as the primer. The amplified products were sequenced and analyzed by the NCBI BLAST algorithm tool. The phylogenetic analysis was conducted by MEGA 5.0 based on maximum likelihood algorithm.

### Lignin degradation

To prepare fungus seed cultures, an agar square from fully grown mycelia on PDA medium was inoculated in 100 mL of basic medium contained (g/L) glucose 20.0, peptone 1.0, yeast extract 2.0, (NH_4_)_2_SO_4_ 4.0, KH_2_PO_4_ 1.0, K_2_HPO_4_ 1.0, CaCl_2_ 0.3 g/L, MgSO_4_ 0.3, NaCl 0.1, and incubated at 28 °C for 7 days with shaking at 180 rpm. The seed cultures were centrifuged at 4000 rpm for 5 min to collect the fungus pellets. The collected pellets were washed three times with 0.9% NaCl to remove the cultures. Then the washed pellets were inoculated into lignin medium contained (g/L) (NH_4_)_2_SO_4_ 4.0, KH_2_PO_4_ 1.0, K_2_HPO_4_ 1.0, NaCl 0.1, MgSO_4_ 0.3, MnSO_4_ 0.05, FeSO_4_ 0.01, CuSO_4_ 0.01, ZnSO_4_ 0.01, CoCl_2_ 0.005, Na_2_MoO_4_ 0.001, KAl(SO_4_)_2_ 0.001, H_3_BO_3_ 0.01, alkaline lignin (from Norway spruce (*Picea abies*) wood) (CAS No. 8068-05-1, Catalog number 370959, Sigma-Aldrich) 3.0, and incubated at 28 °C with shaking at 180 rpm for 13 days. Cultures containing alkaline lignin without inoculating fungus strain were used as control and incubated in the same conditions as the fungal treatment. The culture sample was centrifuged at 12,000 rpm for 10 min, and the collected pellets were washed twice with deionized water to elute the absorbed lignin on the surface of fungus mycelium. The washed cell pellets were collected and dried at 45 °C for 48 h for mycelial biomass dry weight assay. The supernatant and eluent were collected and lyophilized completely. The dried powder was weighed for degradation rate calculation as the following formula:

$$ {\text{Lignin degradation rate }}\left( \% \right)\, = \,\left[ {\left( {M_{0} - M_{{\text{n}}} } \right)/M_{0} } \right]\, \times \,100\% , $$

where M_0_ is the initial lignin weight, and M_n_ is the nth day sampling lignin residue weight.

The decolorization of the lignin culture sample was assayed according to the standard method of the Canadian Pulp and Paper Association [55]. All assays were performed with three replicates.

### Ligninolytic enzyme assay

Laccase (Lac) activity was detected by using 2,2,-azino-bis(3-ethylbenzothiazoline-6-sulfonic acid) (ABTS) as substrate and monitoring the production of ABTS radical at 420 nm (ε420 = 36,000 M^−1^ cm^−1^) according to the method of Nakagawa et al. [56]. Lignin peroxidase (LiP) activity was assessed by monitoring oxidation of veratryl alcohol to veratraldehyde at 310 nm (ε310 = 9300 M^−1^ cm^−1^) based on the method of Kirk et al. [57]. Manganese peroxidase (MnP) activity was assayed by monitoring oxidation of 2,6-dimethyl phenol to coerulignone at 469 nm (ε469 = 49,600 M^−1^ cm^−1^) according to method of Wariishi et al. [58]. The amount of enzyme needed to produce 1 μM product per minute under the assay conditions was defined as one unit of enzyme activity.

### Secretory proteins extraction and identification

The culture samples of fungus strains incubated in lignin-containing medium was withdrawn at 7th day and centrifuged at 12,000 rpm, 4 °C for 10 min. The collected supernatant was filtered through 0.45-μm membranes and was lyophilized completely. The dried powder was resuspended in a buffer containing 8 M urea, 4% (w/v) 3-[(3-cholamidopropyl) dimethylammonio] propanesulfonate, 40 mM dithiothreitol. Protein concentration was determined by a non-interference protein assay kit (Sangon Bio-tech, Shanghai, China). The peptide obtained by trypsin digestion was subjected to nano-liquid chromatography–tandem mass spectrometry (nanoLC–MS/MS). NanoLC–MS/MS analysis was performed on an Orbitrap Fusion Tribrid MS (Thermo, Waltham, MA, USA) equipped with an UltiMate 3000 system according to the procedure described in our previous work [59]. The obtained raw MS/MS data were processed by Maxquant proteomics software (Version 1.6.4) against UniProt proteomes of *Trametes* genus. The parameters used for peptide identification was set as described in our previous work [59].

### GC–MS analysis

The cultures used for GC–MS analysis were pretreated as previously described [60]. The procedure of GC–MS test was performed as described by Chen et al. [40]. The obtained mass spectra were compared with that of the NIST library available in the instrument and the retention time to the original standards.

### FTIR analysis

The chemical structure changes of lignin before and after treated by strain were investigated by FTIR analysis. Samples were ground with dry KBr prior to test. FTIR analysis was conducted on A Varian 2000 FTIR spectrometer range from 4000 to 400 cm^−1^ at 4 cm^−1^ spectral resolution for 32 scans.

### SEM analysis

The collected lignin samples were freeze-dried, then covered with gold to prevent buildup of static charges. SEM analysis was carried out with a JEOL JSM-7900F scanning electron microscopy (Tokyo, Japan).

### 2D-HSQC NMR analysis

Approximately 50 mg of dried lignin powder was dissolved in 0.5 mL of dimethyl sulfoxide (DMSO)-*d*_6_ in an NMR tube. To promote the lignin solubility, the NMR tube was dipped into an ultrasonic bath for 3 h. 2D-HSQC NMR analysis was conducted on a Bruker AVANCE 400 MHz NMR spectrometer (Bruker BioSpin, Rheinstetten, Germany). The spectra were collected at 25 °C using a Bruker standard pulse sequence “hsqcetgpsisp2”. The procedure was performed as described by Ma et al. with slight modification [45]. The spectral widths of ^1^H and ^13^C were 5000 Hz and 18,000 Hz, respectively. A total of 2048 collected complex points with 1.5 s recycle delay were acquired for ^1^H dimension. The number of transients was 64 for ^13^C dimension with 256 time increments. The ^1^J_CH_ was set to 145 Hz. HSQC correlation peaks were assigned by comparison with the literature reports [37, 61–64]. Semiquantitative analysis of volume intergrals of HSQC correlation peaks was conducted by MestReNova 11.0.4 as described by Ma et al. [45].

### Py-GC/MS analysis

Analytical pyrolysis coupled to gas chromatography with mass spectrometeric detection was conducted as previously described with some modification [60]. Briefly, pyrolysis was performed at 500 °C for 1 min on PY-3030D pyrolyzer (Frontier Lab, Japan) coupled with a gas chromatography/mass spectrometer (7890B-5977A, Agilent Technologies, Germany). The chromatography was programmed from 40 °C to 150 °C at 5 °C/min, followed by 10 °C/min to 280 °C, and then held for 20 min. The chromatographic signals were identified by comparing the experimental mass spectrum with those reported in the National Institute of Standards and Technology (NIST) library. The relative abundance of each identified pyrolysate was calculated as the percentage of the sum of all peak areas.

### Statistical analysis

The statistical analysis was performed by Origin 9.0 software, and the experimental data were presented as the replicate mean ± standard deviation (SD).

## Supplementary Information

**Additional file 1: Figure S1.** Detection of colorization and decolorization zone by *T*. *hirsuta* X-13 on guaiacol-containing (a and b) and Azure B-containing (c and d) PDA medium. A and C, control; B and D, inoculated with *T*. *hirsuta* X-13.

**Additional file 2: Figure S2.** Morphologic analysis of *T*. *hirsuta* X-13. (a) Colony morphology of *T*. *hirsuta* X-13 grown on PDA medium plate. (b) Microscopic photograph of *T*. *hirsuta* X-13 mycelium.

**Additional file 3: Table S1.** The percentage of main components in alkaline lignin.

**Additional file 4: **The identified proteins from secretome of *T*. *hirsuta* X-13 in lignin-containing medium.

**Additional file 5: **Functional classification of identified proteins from secretome of *T*. *hirsuta* X-13.

**Additional file 6: Figure S3.** Distribution of the identified proteins based on functional classification.

**Additional file 7: Table S2.** Identification of proteins involved in lignin degradation in the secretome of *T*. *hirsuta* X-13 in lignin-containing medium.

**Additional file 8: Figure S4.** The total ion chromatograms of the control (a) and lignin samples treated with *T*. *hirsuta* X-13 for 13 days (b).

**Additional file 9: Table S3.** The identified pyrolysates from lignin samples before and after treated by *T*. *hirsuta* X-13 at different time with relative peak areas.

## Data Availability

All data generated or analyzed during this study are included in this published article and its supplementary information file.

## Abbreviations

FTIR: Fourier transform infrared spectroscopy
GC–MS: Gas chromatography–mass spectrometry
SEM: Scanning electron micrographs
2D-HSQC NMR: Two-dimensional heteronuclear single quantum coherence NMR
PDA: Potato dextrose agar
ITS: Internal transcribed region sequence
Lac: Laccase
LiP: Lignin peroxidase
MnP: Manganese peroxidase
DMSO: Dimethyl sulfoxide
SD: Standard deviation
CGMCC: China General Microbiological Culture Collection
G: Guaiacyl group
S: Syringyl group
H: *p*-Hydroxyphenyl group
pCA: *p*-Coumarates
FA: Ferulate
py-GC–MS: Pyrolysis gas chromatography–mass spectrometry
