# Supplementary figures and images for: Elucidation of ligninolysis mechanism of a newly isolated white-rot basidiomycete Trametes hirsuta X-13
Source: Biotechnol Biofuels. 2021 Sep 25;14:189. doi: 10.1186/s13068-021-02040-7 (PMC8466896; doi:10.1186/s13068-021-02040-7)

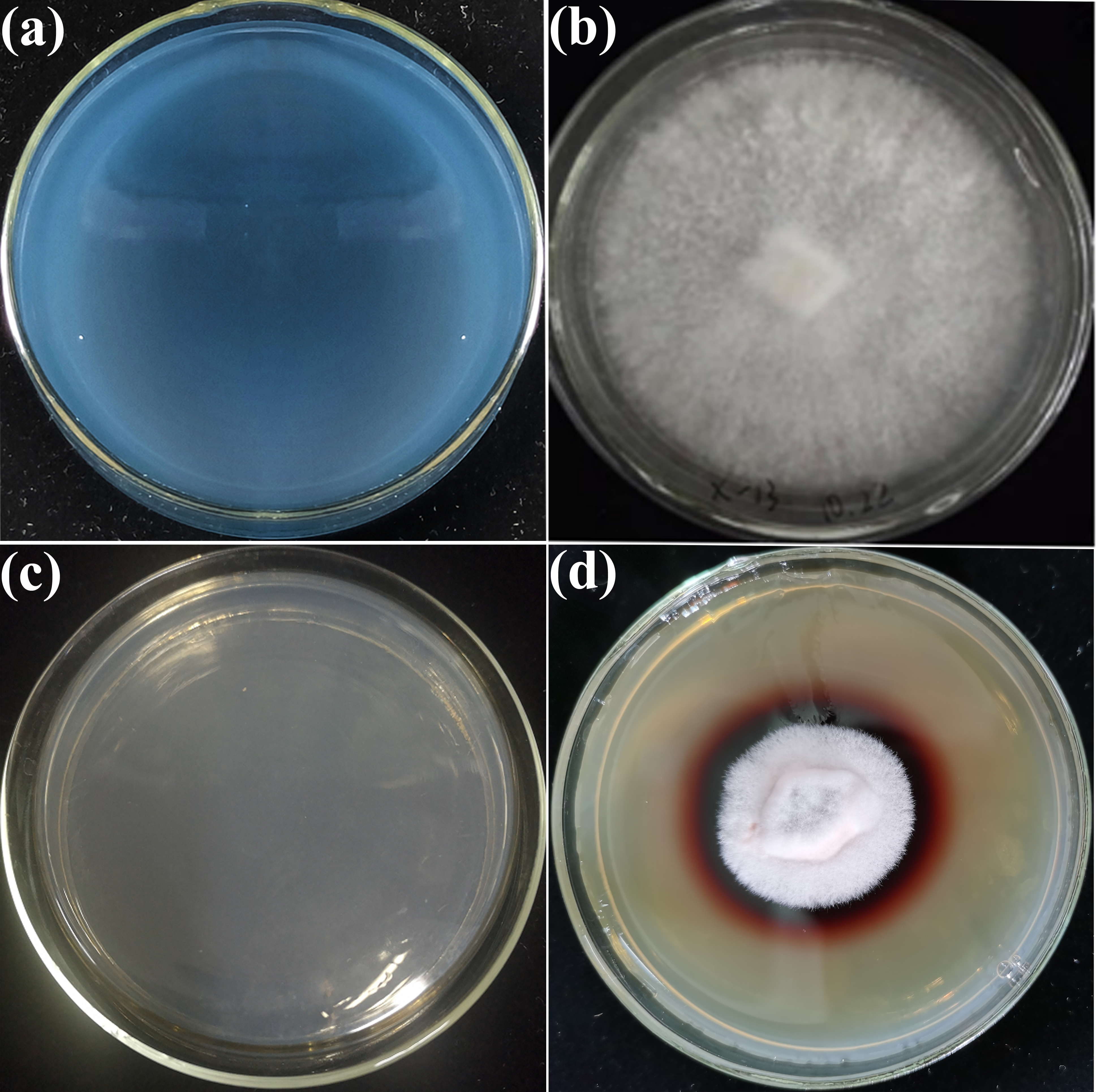

Supplement: Supplementary file 1 — Additional file 1: Figure S1. Detection of colorization and decolorization zone by T. hirsuta X-13 on guaiacol-containing (a and b) and Azure B-containing (c and d) PDA medium. A and C, control; B and D, inoculated with T. hirsuta X-13. [file 13068_2021_2040_MOESM1_ESM.png]

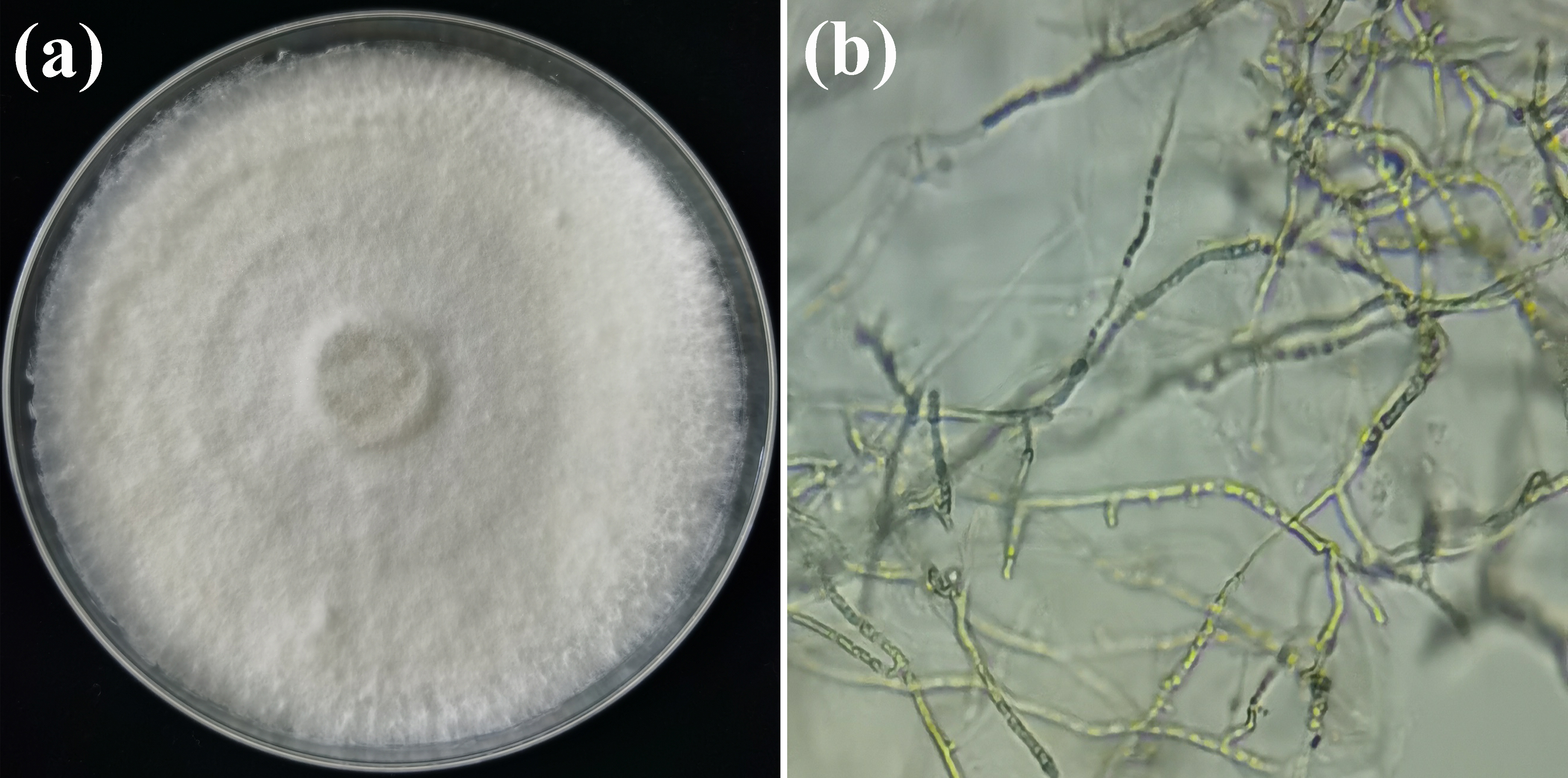

Supplement: Supplementary file 2 — Additional file 2: Figure S2. Morphologic analysis of T. hirsuta X-13. (a) Colony morphology of T. hirsuta X-13 grown on PDA medium plate. (b) Microscopic photograph of T. hirsuta X-13 mycelium. [file 13068_2021_2040_MOESM2_ESM.png]

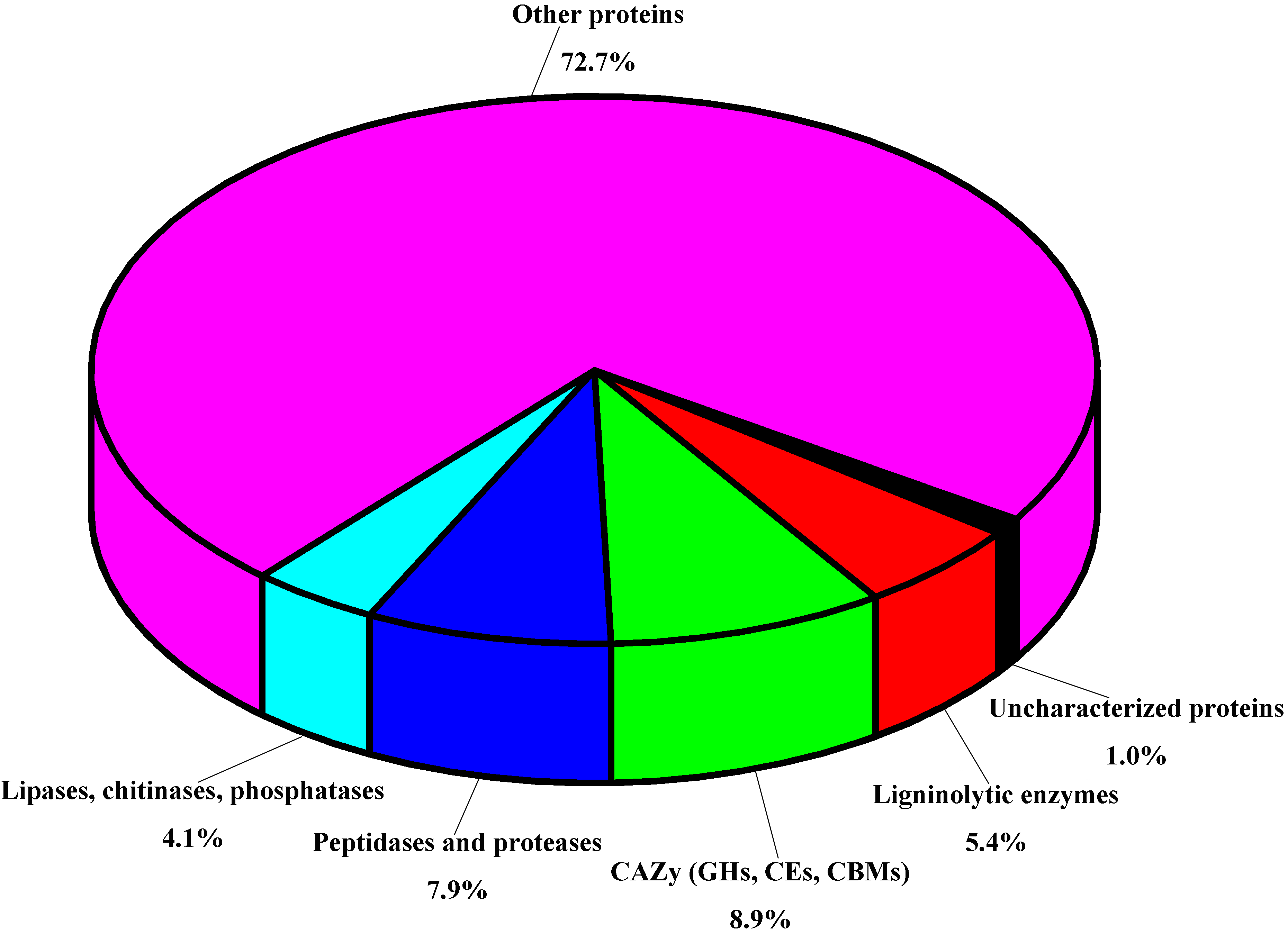

Supplement: Supplementary file 6 — Additional file 6: Figure S3. Distribution of the identified proteins based on functional classification. [file 13068_2021_2040_MOESM6_ESM.png]

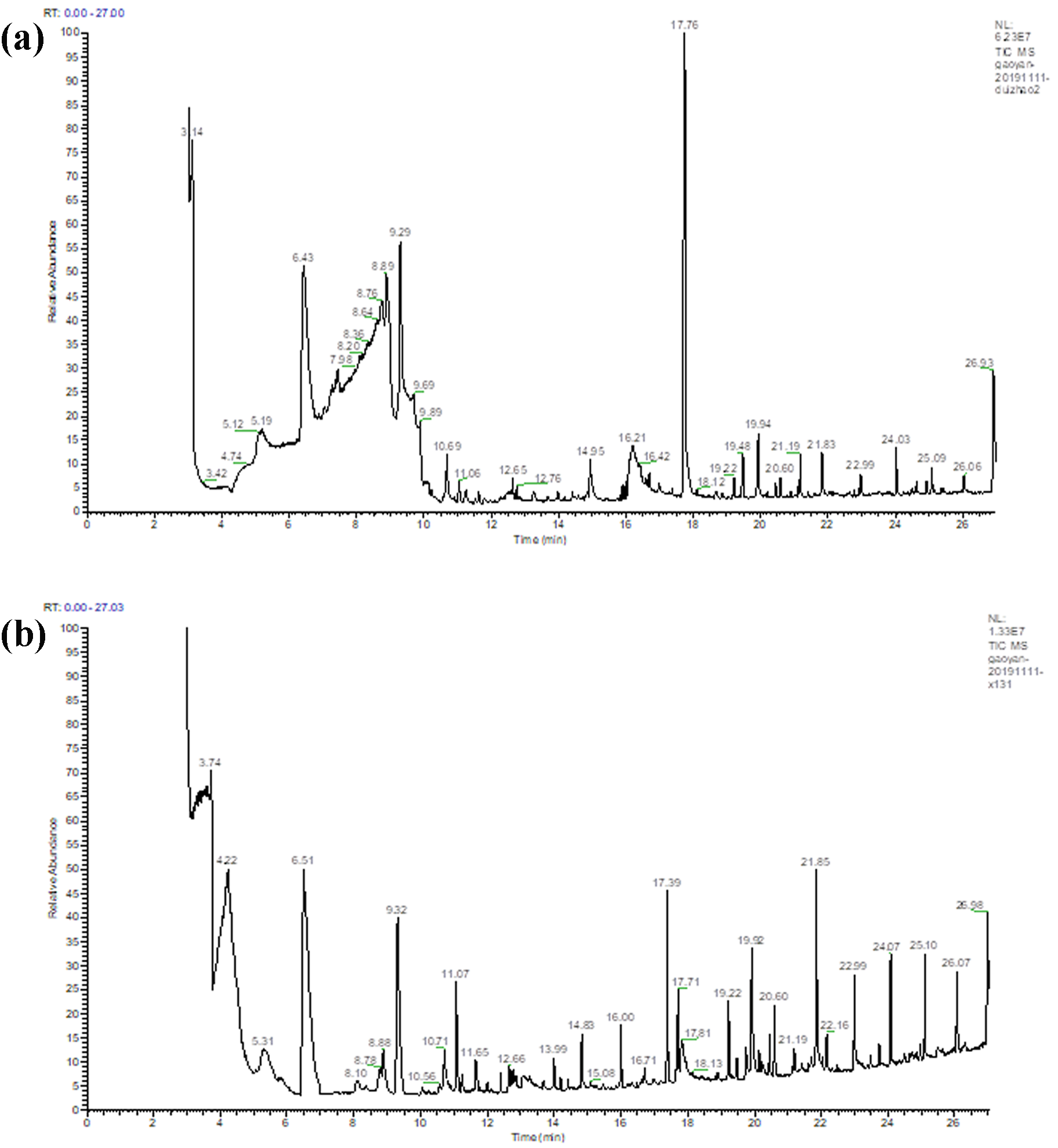

Supplement: Supplementary file 8 — Additional file 8: Figure S4. The total ion chromatograms of the control (a) and lignin samples treated with T. hirsuta X-13 for 13 days (b). [file 13068_2021_2040_MOESM8_ESM.png]
