# Supplementary material for: Elucidation of ligninolysis mechanism of a newly isolated white-rot basidiomycete Trametes hirsuta X-13
Source: Biotechnol Biofuels. 2021 Sep 25;14:189. doi: 10.1186/s13068-021-02040-7 (PMC8466896; doi:10.1186/s13068-021-02040-7)
Supplement: Supplementary file 3 — Additional file 3: Table S1. The percentage of main components in alkaline lignin. [file 13068_2021_2040_MOESM3_ESM.pdf]

**Table S1** The percentage of main components in commercial alkaline lignin

| **Components** | **Percentage (%)** |
|---|---|
| Lignin | 94.61 |
| Glucose | 0.24 |
| Xylose | 0.85 |
| Arabinose | 0.43 |
| Ash | 2.80 |
| Others | 1.07 |
