# Supplementary material for: Elucidation of ligninolysis mechanism of a newly isolated white-rot basidiomycete Trametes hirsuta X-13
Source: Biotechnol Biofuels. 2021 Sep 25;14:189. doi: 10.1186/s13068-021-02040-7 (PMC8466896; doi:10.1186/s13068-021-02040-7)
Supplement: Supplementary file 7 — Additional file 7: Table S2. Identification of proteins involved in lignin degradation in the secretome of T. hirsuta X-13 in lignin-containing medium. [file 13068_2021_2040_MOESM7_ESM.pdf]

Table S2 Identification of proteins involved in lignin degradation in the secretome of *T. hirsuta* X-13 in lignin-containing medium

**Accession number**	**Protein description**	**Coverage (%)**	**Peptides**	**Unique peptides**	**MW (kDa)**	**Subcellular localization prediction**
A0A2Z2CBW5	Laccase	38.8	13	3	55.7	Extracellular
A0A2Z2CDY7	Laccase	20.4	5	5	56.316	Extracellular
Q5MBH5	Laccase	29.5	10	4	53.963	Extracellular
A0A6M5CX58	Laccase	17.3	6	5	55.827	Extracellular
Q99055	Laccase	5.6	2	1	55.643	Extracellular
Q308Q9	Laccase	3.6	1	1	57.011	Extracellular
A0A1M2V5W2	Manganese peroxidase	7.5	3	1	38.149	Extracellular
A0A1M2V5W5	Manganese peroxidase	15.8	7	1	37.985	Extracellular
A0A1M2VYS0	Manganese peroxidase	10.1	6	3	73.323	Extracellular
A0A1M2VZP3	Manganese peroxidase	14.7	6	1	37.877	Extracellular

A0A1M2W058	Manganese peroxidase	9	3	1	37.83	Extracellular
A0A1M2W3Y9	Manganese peroxidase	15.5	6	2	38.35	Extracellular
Q6B6N0	Manganese peroxidase	17.6	7	2	38.344	Extracellular
Q6B6N2	Manganese peroxidase	12.6	7	2	38.141	Extracellular
A0A1M2V504	Lignin peroxidase	17.3	5	1	38.919	Extracellular
A0A1M2W3T4	Lignin peroxidase	19.2	5	1	38.731	Extracellular
A0A1M2V8Q1	Lignin peroxidase	21.8	6	1	39.709	Extracellular
A0A1M2W425	Lignin peroxidase	8.5	3	2	39.303	Extracellular
A0A1M2VCM9	Dye-decolorizing peroxidase	2.9	1	1	52.232	Cytoplasm
A0A1M2VCF4	Dyp-decolorizing peroxidase	3.5	1	1	53.409	Nucleus
A0A1M2V6U7	Versatile peroxidase VPS1	3.6	2	2	40.609	Extracellular

A0A1M2VBV7	Versatile peroxidase VPL1	19.2	7	5	38.411	Extracellular
A0A1Y2IJI7	Peroxidase	11.4	4	1	39.343	Extracellular
A0A1Y2IJJ3	Peroxidase	10.4	3	1	38.352	
A0A1M2VIL4	Peroxidase	8.5	2	1	41.488	Mitochondria
A0A1Y2IML5	GMC oxidoreductase	25.2	12	2	72.34	Cytoplasm
A0A1M2V6J4	Copper radical oxidase	11.2	5	5	81.513	Extracellular
A0A1M2VRW6	Copper radical oxidase	13.4	7	4	59.19	Extracellular
A0A1Y2IBX3	Copper radical oxidase	5.6	3	1	59.445	Plasma membrane
A0A1Y2J577	Copper radical oxidase	2.2	1	1	83.514	Extracellular
A0A1M2VVR1	Copper radical oxidase	9.8	6	2	97.904	Extracellular
A0A1M2W1C9	Glucose oxidase	8.8	5	2	59.535	Extracellular
A0A1M2W1F4	Glucose oxidase	8.2	5	2	64.51	Extracellular
A0A1M2W6Y0	Glucose oxidase	2.1	1	1	65.196	Extracellular

A0A1M2VRY4	Glyoxal oxidase	12.8	6	1	59.799	Extracellular
A0A1Y2I779	Cytochrome P450 monooxygenase	3.8	1	1	37.677	Plasma membrane
A0A1Y2IR54	Cytochrome P450 monooxygenase	2.5	1	1	58.46	Plasma membrane
A0A1M2VNB4	Lytic polysaccharide monooxygenase	13.5	2	2	19.96	Extracellular
A0A1M2V359	Glutathione transferase omega class	3.5	1	1	29.527	Cytoplasm
A0A1M2V5E0	Glutathione S-transferase	8.3	2	1	26.169	Cytoplasm
A0A1M2VLW9	Glutathione S-transferase	21.5	3	2	25.97	mitochondria
A0A1M2W0W3	Glutathione S-transferase	11.2	2	1	28.803	Cytoplasm
