# Supplementary material for: Elucidation of ligninolysis mechanism of a newly isolated white-rot basidiomycete Trametes hirsuta X-13
Source: Biotechnol Biofuels. 2021 Sep 25;14:189. doi: 10.1186/s13068-021-02040-7 (PMC8466896; doi:10.1186/s13068-021-02040-7)
Supplement: Supplementary file 9 — Additional file 9: Table S3. The identified pyrolysates from lignin samples before and after treated by T. hirsuta X-13 at different time with relative peak areas. [file 13068_2021_2040_MOESM9_ESM.pdf]

Table S3 The identified pyrolysates from lignin samples before and after treated by *T. hirsuta* X-13 at different time with relative peak areas.

Rention time (min)	Compound	Group	Peak area (%)		
			Control	7 d	13 d
5.2	Phenol	H	1.95	1.67	1.47
6.4	2-Methylphenol	H	2.68	1.84	1.72
8.7	4- Methylphenol	H	1.07	1.25	1.48
8.9	Guaiacol	G	2.43	3.46	2.63
9.6	1,2-Benzenediol	H	1.86	1.42	1.35
10.3	Methlguaiacol	G	2.47	2.87	2.67
10.6	2,6-Dimethylphenol	H	2.33	1.89	2.08
10.8	4-Ethylphenol	H	2.51	1.23	1.32
10.9	4-Ethylguaiacol	G	3.97	3.26	2.76
11.1	1,2-Benzenediol, 4-methyl	G	3.17	3.44	2.71
11.6	1,4-Benzenediol, 2-methoxy-	G	1.95	1.21	2.43
11.8	4-Methyl-benzaldehyde	G	2.76	2.03	1.84
11.9	4-Vinylguaiacol	G	1.3	2.84	2.43
12.2	Eugenol	G	0.89	2.63	1.74
12.4	Vanillin	G	1.23	3.68	1.55
12.6	2-Methoxy-4-vinylphenol	G	2.29	1.65	1.52
12.8	3-Methoxy-5-methylphenol	H	1.33	1.96	1.61

12.9	4-Methylsyringol	S	0.28	0.87	0.77
13.2	4-Propylguaiacol	G	2.34	1.87	2.07
13.3	3-Methoxy-2-benzenediol	H	2.29	1.35	1.15
13.5	Catechol		1.88	2.31	2.27
13.8	Syringol	S	1.49	1.64	1.76
14.1	3,4-Dimethoxyphenol	S	0.71	1.37	1.64
14.3	2,6-Dimethoxytoluene	S	ND	0.46	0.67
14.4	4-Vinylsyringol	S	1.46	0.18	1.3
14.5	Isoeugenol	G	3.46	2.57	2.43
14.7	3-Methoxy-4,5,6-trimethylphenol	S	1.85	1.38	ND
14.9	Benzene, 1,2,3-trimethoxy-5-methyl-	S	0.62	ND	0.97
15.0	Benzene, 1,2-dimethoxy-4-(2-propenyl)-	G	2.23	2.04	1.59
15.2	Phenol, 2,6-dimethoxy-4-(2-propenyl)-)	G	2.51	1.95	1.64
15.4	3,4,5-Trimethoxybenzaldehyde	S	1.74	1.57	1.35
15.5	Phenol, 2-methoxy-4-propyl	G	2.49	1.13	1.48
15.8	1,2,3-Trimethoxy-5-methylbenzene	S	1.43	0.72	1.46
15.9	Vanillic acid	G	3.26	3.06	2.78

16.3	4-Hydroxy-2-methoxycinnamaldehyde	S	1.69	0.52	0.84
16.8	1,2-Dimethoxy-4-(1-methoxyethenyl)benzene	G	ND	2.26	1.21
17.2	1,2-Dimethoxyl-4-(3-methoxy-1-propenyl)-benzene	G	0.52	0.34	2.17
17.4	2-Propanone, 1-(4-hydroxy-3-methoxyphenyl)-	G	2.82	1.82	1.46
17.8	Isovanillic acid	G	2.41	nd	1.89
18.1	Methoxyeugenol	G	4.38	3.47	3.26
18.6	1,2-Benzenedicarboxylic acid	H	3.64	2.88	2.49
18.8	Coniferaldehyde	G	2.93	3.01	2.35
19.3	3,5-Dimethoxy-4-hydroxycinnamaldehyde	S	1.87	1.64	1.66
20.4	2-Propenal, 3-(4-methylphenyl)	H	0.86	0.77	0.62
22.6	4-((1E)-3-Hydroxy-1-propenyl)-2-methoxyphenol	G	1.98	1.51	1.33
23.9	4-Hydroxy-3,5-dimethoxyallylbenzene	S	1.71	1.04	1.42
	Total peak areas of H group		17.52	16.26	15.29
	Total peak areas of G group		57.07	52.1	47.94

Total peak areas of S group	15.16	11.39	13.84
S/G	0.26	0.22	0.29
